# A seed and soil model of gut dysbiosis in Alzheimer’s disease

**DOI:** 10.18632/aging.204840

**Published:** 2023-06-19

**Authors:** Chun-Che Hung, Kristi M. Crowe-White, Ian M. McDonough

**Affiliations:** 1 Department of Occupational Therapy and Graduate Institute of Behavioral Sciences, Chang Gung University, Taiwan; 2 Department of Human Nutrition and Hospitality Management, The University of Alabama, Tuscaloosa, AL 35487, USA; 3 Alabama Research Institute on Aging, The University of Alabama, Tuscaloosa, AL 35487, USA; 4 Department of Psychology, The University of Alabama, Tuscaloosa, AL 35487, USA

**Keywords:** aging, neurocognitive disorders, gut-brain axis, gut microbiota, dysbiosis

Alzheimer’s disease (AD) is an irreversible neurodegenerative disorder characterized by persistent neuropathological changes in the brain with progressive deterioration of memory and other cognitive domains. AD limits an individual’s function to participate in valuable occupations and decreases the quality of life of both patients and their caregivers. As a result, AD is considered an urgent public health concern in the medical and scientific communities. Despite decades of research, the underlying etiology of AD remains unclear, with no available therapeutic strategies to prevent or stop AD progression. However, recent research has demonstrated a crucial role of gut microbiota in the etiopathogenesis of AD that offers a new window into possible origins and consequences of AD through interactions between gut microbiota and the central nervous system, known as the “microbiota-gut-brain axis” [1].

The human gut microbiota is a bidirectional ecosystem with multiple feedback loops comprised by a dynamic and complex microbial community incorporating over 1,000 species and 7,000 strains of bacteria [2]. The gut microbiota is critical for host protection against pathogens, immune development, and metabolism of dietary nutrients and drugs. In healthy individuals, the gut microbial composition is established early in life and remains relatively stable over time. Nevertheless, this ecosystem may become destabilized as a result of aging, environmental factors, and lifestyle habits such as one’s diet. Shifts in gut microbial composition and diversity (i.e., gut dysbiosis) have been reported to influence neuroimmune and neuroendocrine functions through a bottom-up fashion resulting in neuroinflammation, microglial dysregulation, and aberrant protein aggregation in the AD brain. Accordingly, this dysbiotic condition may set the stage for a toxic brain environment that stimulates AD neuropathophysiology, including the deposition of amyloid-beta (Aβ) plaques and neurofibrillary tangles.

The relationship between disruptions in the microbiota-gut-brain axis and AD can be explained by the Seed and Soil Model of Neurocognitive Disorders [3] (Figure 1). Based on this model, the “seed” represents a predisposition to a neurocognitive disorder (e.g., genetic profile) and the “soil” refers to factors that moderate the expression of that seed. Together, the seed and the soil ultimately determine whether a person will develop the disorder. This model was created to explain why some people who are predisposed to develop neurocognitive disorders do not develop them. Although this model did not originally apply to the microbiota-gut-brain axis, the concept is general enough that it can be applied to many new contexts as ideas evolve. In the case of AD and the microbiota-gut-brain axis, the seed could represent a polygenic risk score or family history of AD, whereas the soil could be represented by certain dysbiotic taxa. Dysbiotic taxa can contribute to many sequalae including altered intestinal permeability that leads to a leaky gut and fosters the activation of local and distant immune cells [2]. Given that the metabolites of gut leakiness are linked to increased permeability of the blood-brain barrier, these dysfunctions promote the translocation of bacterial endotoxins from the gut to the brain and increase inflammation within the system. According to the Seed and Soil Model of Neurocognitive Disorders, this translocation would create a toxic microenvironment in the brain vulnerable to pathogenesis, especially for those with a genetic predisposition to AD. Consistent with this notion, a recent systematic meta-analysis showed that individuals with AD exhibited less gut microbial diversity than those with mild cognitive impairment (MCI) or healthy controls [1]. Likewise, the gradient changes of abundance from normal cognition to MCI and AD stage were observed in several strains of gut microbiota (i.e., phylum *Proteobacteria*, family *Clostridiaceae*, and genus *Phascolarctobacterium*). Prior evidence also has revealed that gut-derived lipopolysaccharide (endotoxin) acts as an Aβ fibrillogenesis promoter, potentially leading to neuroinflammation and neurodegeneration [4].

**Figure 1:**
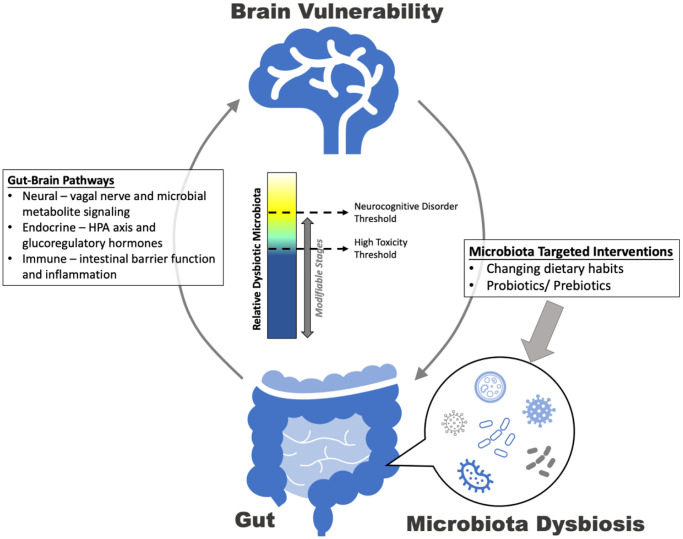
A seed and soil model of gut dysbiosis.

The Seed and Soil Model of Neurocognitive Disorders also proposes that neuroimaging techniques can provide early insight into factors that drive brain alterations related to AD, including the effects of the gut microbiota. For example, using resting-state functional magnetic resonance imaging, gut microbial diversity has been associated with aberrant functional connectivity both within and between multiple brain networks in healthy participants [5]. Another study in healthy young adults also revealed a strong relationship between gut microbial diversity and multimodal neuroimaging measures across multiple brain networks [6]. In both studies, brain alterations mediated the association of gut microbial diversity with cognition, highlighting the key role of the gut microbiota in the normal physiological function of the microbiota-gut-brain axis.

According to the Seed and Soil Model of Neurocognitive Disorders, environmental and behavioral patterns can influence the balance of neuroprotection vs. toxicity of the brain’s microenvironment. Similarly, changing dietary patterns to target gut microbiota composition has been suggested as a potential intervention to improve brain declines associated with the AD trajectory [7]. Specific foods and dietary habits can affect the structure and abundance of different strains of microbiota in the intestine, which is implicated in the maintenance of human homeostasis, more generally. As an example, a recent study has reported that AD patients with better global cognition had more ingestion of coffee or tea [8]. Furthermore, the Mediterranean diet pattern and high intake of plant-based foods, especially those rich in polyphenols might delay an AD diagnosis and increase the levels of fecal short chain fatty acids and bile acids [7]. These lifestyle modifications might facilitate gut homeostasis and ameliorate the dysbiotic events that decrease the brain’s vulnerability to pathogenesis. Without the proper gut environment to foster homeostasis and symbiosis, dysbiotic microbiota drive the disease relationship and development.

In conclusion, the Seed and Soil Model of Neurocognitive Disorders provides a useful explanatory framework to understand multiple varying causal pathways to neurocognitive disorders. Here, we extend the model to better understand how the microbiota-gut-brain axis may play a causal role in the development of AD. However, more research is needed to test additional hypotheses of the model. For example, the model predicts that the combination of both a genetic predisposition and poor lifestyle habits, especially dietary habits, creating gut dysbiosis are necessary conditions to create a brain vulnerable to pathology. It also predicts that early brain alterations likely would take the form of increases in brain activity or within-network connectivity in core networks involved in AD followed by a decrease in activity and connectivity as AD neuropathophysiology progresses.

## References

[1] Hung CC, et al. Aging (Albany NY). 2022; 14:477–96. 10.18632/aging.203826 35027502 PMC8791218

[2] Tarawneh R, et al. Neurosci Biobehav Rev. 2022; 141:104814. 10.1016/j.neubiorev.2022.104814 35934087 PMC9637435

[3] McDonough IM, et al. Aging Ment Health. 2019; 23:793–9. 10.1080/13607863.2018.1531383 30449142

[4] Liu P, et al. Brain Behav Immun. 2019; 80:633–43. 10.1016/j.bbi.2019.05.008 31063846

[5] Cai H, et al. Hum Brain Mapp. 2021; 42:3088–101. 10.1002/hbm.25419 33739571 PMC8193524

[6] Zhu J, et al. Prog Neuropsychopharmacol Biol Psychiatry. 2022; 113:110468. 10.1016/j.pnpbp.2021.110468 34736997

[7] Liu S, et al. Mol Neurobiol. 2020; 57:5026–43. 10.1007/s12035-020-02073-3 32829453 PMC7541367

[8] Hsiao HT, et al. Nutrients. 2022; 14:5300. 10.3390/nu14245300 36558459 PMC9784891

